# COVID-19 vaccine communication and advocacy strategy: a social marketing campaign for increasing COVID-19 vaccine uptake in South Korea

**DOI:** 10.1057/s41599-023-01593-2

**Published:** 2023-03-16

**Authors:** Shin-Ae Hong

**Affiliations:** Crisis, Disaster and Risk Management, Sungkyunkwan University, Suwon, South Korea

**Keywords:** Social policy, Health humanities

## Abstract

Research evidence suggests that communication is a powerful tool for influencing public opinion and attitudes toward various health-related issues, such as vaccine reluctance, provided it is well-designed and thoughtfully conducted. In particular, social marketing techniques that alter the target audience’s behaviors for the public good can substantially improve vaccine uptake if adopted as a communication strategy in immunization programs to counter public hesitancy. This study presents evidence from the Korean government’s current coronavirus disease 2019 (COVID-19) vaccination campaign, which successfully applied a social marketing approach. By the end of August 2022, South Korea had achieved high vaccine coverage, with 94.8% of the population (12+) receiving a second dose, 71.3% a third dose, and a fourth dose drive currently underway. There are five crucial factors to consider when preparing official communication for an immunization program: (i) a high degree of proactiveness, (ii) credibility, (iii) fighting misinformation, (iv) emphasizing social norms and prosocial behavior, and (v) coherence. Although using social marketing strategies may not be successful in all circumstances, the lessons learned and current implementation in Korea suggest their efficacy in fostering vaccine acceptance. This study offers valuable insights for government agencies and global public health practitioners to develop effective targeted campaign strategies that enhance the target population’s vaccination intention.

## Introduction

The unprecedented spread of severe acute respiratory syndrome coronavirus 2 (SARS-CoV-2) has resulted in the century’s first major public health and economic crisis.

Two years after the pandemic, the world experienced several deadly waves of the coronavirus disease-2019 (COVID-19). Governments worldwide have hurried to implement mass vaccine rollout programs, but public acceptance of the vaccine is crucial for long-term COVID-19 control and prevention. Vaccination is generally regarded as a critical and cost-effective protective intervention for public health (Polack et al., 2020; Anderson et al., 2020; WHO, 2021a). While vaccines are the epicenter of the global response to the pandemic, public health officials worldwide are encountering growing vaccine hesitancy, significantly impeding their efforts for reaching herd immunity. Vaccine hesitancy or refusal can be affected by numerous factors such as availability, confidence, cost, anxiety, convenience, and misinformation (Nguyen et al., 2021; Loomba et al., 2021). Additionally, poor public health communication can cause confusion, skepticism, and resistance in the population, which may negatively impact the implementation of immunization programs (Butler et al., 2015).

There is a need for locally contextualized research that closely examines effective immunization campaign practices. It is important to identify useful principles, approaches, and strategies for effective mass vaccination campaigns that increase uptake and address specific aspects influencing vaccine hesitancy. The author reviews a few lessons learned from public vaccination campaigns in South Korea (hereinafter Korea), which earned global acclaim for their effective and successful COVID-19 immunization programs. By August 2022, 94.8% of Korean citizens over 12 years had already received their second dose, 71.3% had received the third (booster) dose, and 16.2% (individuals over 50 years) had received the fourth dose (Korean Ministry of Health and Welfare [KMHW], 2022a). These exemplary statistics are indicative of Korea’s high vaccination rate compared to the rest of the world. From the time vaccines became available for public use until August 2022, Korea administered 248.88 vaccine doses per 100 people. Meanwhile, China administered 240.75 vaccine doses per 100 people, Italy 236.93, Canada 232.32, Australia 225.29, the United Kingdom 224.60, France 221.56, Germany 221.44, Israel 196.12, and the United States 183.73 (Our World in Data, 2022). Before implementing the COVID-19 vaccination campaign, the Korean public largely showed strong hesitancy, having a “wait and see” attitude toward the COVID-19 vaccine; many were concerned about the adverse effects of vaccination (You, 2021; Views and News, 2021). However, the country overcame widespread vaccine hesitancy by applying social marketing as a behavioral change strategy to public health campaigns. Korea achieved high immunization coverage by addressing the causes of vaccine skepticism and positively appealing to the target audience’s rationale.

Although there is no catholicon communication method for public health communications in times of unparalleled pathogenic crisis, this study highlights the potential value of commercial marketing techniques in immunization promotion campaigns. Such techniques may bolster a campaign’s chances of success by fostering behavioral changes in the target population. This study introduces evidence from South Korea’s successful COVID-19 immunization campaign, incorporating core elements of commercial marketing techniques to influence people’s behavior. It also identifies five key communication attributes that the country’s public health authority considered for an optimum impact while implementing its campaign: (i) a high degree of proactiveness, (ii) credibility, (iii) fighting misinformation, (iv) emphasizing social norms and prosocial behavior, and (v) coherence. This finding offers new insights that can be employed at a national and global scale and is a notable contribution to the literature (Lefebvre, 2013; Thorpe et al., 2022; Jin et al., 2021; Murewanhema et al., 2022; Boyd and Buchwald, 2022; Hyland-wood et al., 2021).

## Theoretical perspective: social marketing as a public health intervention strategy

Social marketing has attracted significant attention from health researchers and practitioners as an effective and holistic intervention to increase vaccine uptake and respond to vaccine reluctance (Nowak et al., 2015; French and Gordon, 2020). The World Health Organization (WHO) has recommended social marketing strategies to build vaccine confidence and address low vaccination rates (WHO, 2020, 2022a).

Social marketing programs use commercial marketing-based principles to motivate individuals to adopt the suggested social behaviors to achieve common societal interests. Social marketing is defined by the International Social Marketing Association (iSMA) as an activity that “seeks to develop and integrate marketing concepts with other approaches to influence behaviors that benefit individuals and communities for the greater social good” (iSMA, 2020). Social marketing focuses on effecting change at multiple levels of society (person, society, and institution), culminating in transformation (Kemper and Ballantine, 2017). These models have been used widely in the public health field to enhance the health and well-being of people by increasing awareness of health issues and altering health behaviors (e.g., limiting cigarette use, advocating a healthy diet, using contraceptives, HIV prevention) (Kemper and Ballantine, 2017; Shams, 2018). With the release of COVID-19 vaccines, social marketing techniques are adopted in immunization campaigns to educate the general public about the vaccine’s safety and effectiveness, build positive social norms for vaccine uptake, and “nudge” people toward getting vaccines (Rhodes et al., 2021; Evans and French, 2021).

The core concept of the social marketing program is a willful, voluntary behavioral change for one’s own satisfaction and self-interest (Kotler and Armstrong, 2020). Customer buying behaviors are regarded as value exchanges in which both sides mutually benefit. As both players primarily act in their own interests, commercial marketers must first understand their targeted audience’s underlying needs, wants, and interests for their marketing to be successful. Customer orientation is a key theoretical cornerstone of social marketing programs. Following this assessment, a marketer delivers a product that meets customers’ needs and lowers the barriers that might hinder their purchasing behavior. Customers offset the disadvantages of alternatives and decide whether the marketer’s product or service is beneficial and valuable before voluntarily exchanging their resources for the goods offered (French, 2017).

### The marketing mix: The 4Ps for behavior change

The “4Ps” model—comprising product, price, place, and promotion—is a central element of the social marketing framework. First suggested by Kotler and Zaltman (1971), the model’s four key categories are beneficial for implementing marketing initiatives. The 4Ps help facilitate the development, communication, and promotion of a product to its target audience. Each dimension consists of a marketing variable that aims to make a product, service, or advocated behavior more appealing.

The premise is that a social marketer must produce the right product at the right price, distribute it to the right market in the right location, and market it to the right group (Lefebvre, 2013). The *product* comprises the offering’s qualities and functions, incorporating the benefits of utilizing the offering or engaging in a suggested set of actions. Critical to its desirability is how it corresponds to the consumer’s aspirations, needs, and interests and offers a solution to a problem. The *price* refers to the consumer’s cost or sacrifice in exchange for the product. It involves money, time, and physical or psychological effort invested in the exchange. The *place* is associated with the intermediary physical sales location, facilitating marketer–consumer exchange. The design of the place is crucial, as it provides sufficient incentives for consumers to engage with the product. They may include creating easy, convenient, or accessible outlets for people to engage in exchange. *Promotion* encompasses the marketer’s persuasive communication activities that emphasize product features/benefits, associated prices, and places to buy the offering. A promotional strategy usually comprises promotional activities via public relations, media and advertising, message delivery channels, and special events that influence change (MacDonald et al., 2013).

### The marketing mix for COVID-19 vaccine communication

The social marketing approach offers consumer behavioral insights into vaccines and immunizations. The approach helps develop programs and acquire knowledge about the wants, values, and needs of target individuals whose health behavior changes we aim to influence. Immunization program planning decisions can be made when policymakers understand the benefits and barriers of the inoculation program from the targeted audience’s perspective. This enables them to create a demand for vaccination services in the local community. Well-designed immunization campaigns can certainly boost vaccination coverage by enhancing public trust, alleviating anxiety and fear, and enabling people to connect better with the community and its goals (Lee et al., 2022; Shekhar, 2022).

As the Korean public health authority began the campaign, it projected “herd immunity” as the best way to end the pandemic and recommended that everyone be immunized against SARS-CoV-2 (KDCA, 2021a). This policy encouraged the public to embrace new behaviors (vaccine uptake) and avoid antisocial ones (vaccine delay or not getting vaccinated); however, this policy was not mandatory. It relied on voluntary cooperation because it was presumed that the general population would adopt a behavioral change if its benefits/rewards/social consequences suited their needs. Public health officials have applied the social marketing approach to health communication to positively stimulate vaccine-acceptance behavior in the population. They also tailored their communication strategies and distributed accurate vaccine information via diverse media channels. The 4Ps model is used to explain vaccine communication as follows.

#### Product communication

“Product” refers to what is being sold—in this instance, the vaccine’s benefits. Vaccine intention can be predicted by the perceived benefits of vaccines (Lee et al., 2022). Recommended behaviors include adherence to the vaccination schedule or taking the vaccine; engaging in this behavior facilitates health benefits (Nowak et al., 2015; Wassler et al., 2022). Product communication is applied to vaccines by identifying the behavioral mechanisms that benefit people. These behavioral proposals aim to enhance individual and community health by stimulating the human immune system to produce antibodies and fight against COVID-19 infection. This reduces the incidence of contracting or transmitting the infection, lowers the mortality rate, and decreases the chance of developing severe COVID-19 symptoms among fully vaccinated populations (Dye, 2022; Sadarangani et al., 2021; Kerr et al., 2022).

#### Price communication

“Price” refers to the target group barriers to procuring the vaccine, such as financial cost, inaccessibility, inconvenience, and perceived low vaccine efficacy or safety. Immunization planners must consider interventions to reduce these hurdles, alter public perception, and increase the perceived value of vaccination (WHO, 2021b). For strategic communication, public health officials must highlight the drivers of vaccination (e.g., safety, efficacy, health, and social rewards) that outweigh the perceived risk or cost (unexpected side effects, undesirable social consequences, etc.). The Korean government encouraged the public to be immunized to achieve herd immunity, and the communicated goal was to protect the community from virus infection and return to pre-COVID-19 normalcy. Furthermore, it emphasized that adverse side effects are extremely infrequent and assured the public that they would be compensated in the event of an adverse reaction to the vaccine (KDCA, n.d.-a).

#### Place communication

“Place” denotes where and when people can access the vaccination service (e.g., hospitals, clinics, mass vaccination centers, etc.). Given that herd immunity can only be achieved by substantial public buy-in, the majority of the community must embrace product communication (getting a vaccine). Hence, multiple communication tools have been employed via numerous media channels to communicate with the public, effectively manage information flow, and enhance risk communication.

#### Promotion communication

“Promotion” refers to communication strategies for clear, accurate, and coherent information on immunization plans (CDC, n.d.). This involves providing updated vaccine information and suggested actions through trusted media outlets. This communication strategy leveraged public service advertisements, spokespersons, media outreach, and communication materials to promote vaccinations.

### Integrating behavioral intervention into the marketing mix: value creation and exchange

When changing attitudes and behaviors is insufficient, behavioral science proposes the use of other behavioral intervention tools, such as “nudging” or the default option, in combination with persuasive communication to overcome barriers and influence behavior (Evans and French, 2021; French and Gordon, 2020; Dai et al., 2021). Within the proposed approach, Evans and French (2021) recommend using both incentivizing and disincentivizing elements in social marketing strategies to elicit a behavioral response and maintain behavior change. They suggest four types of behavioral interventions: *Hug, Smack, Shove*, and *Nudge*, to increase the target audience’s vaccine uptake against COVID-19. *Hug* is an active cognitive engagement and positive incentive for adaptation (e.g., offering rewards with financial incentives, vaccine badges, vaccine holidays, and access permits for facilities when you have a “vaccine passport”). *Nudge* is a passive cognitive engagement and positive incentive for adaptation (e.g., setting up default options for the entire populace, using small financial rewards, gift cards, or sending reminder text messages or emails for vaccination). *Smack* is an active cognitive engagement and punishment for non-adaptation (e.g., no access to shopping malls, penalty fines, or dismissal from work in high-risk industries without a vaccine certificate). *Shove* is passive cognitive engagement and punishment for non-adaptation (e.g., multiple polymerase chain reaction (PCR) test requirements before using public facilities if not vaccinated). Together with persuasive communication, Korea employed these schemes in its COVID-19 vaccination policy to encourage vaccine uptake.

## Methods

This study reviewed the relevant literature from immunization inception in January 2021 to August 2022. Four major databases were searched: Web of Science, PubMed, ScienceDirect, and Factiva, using the following keywords: COVID-19 vaccination and recommendation OR public health communication/campaign. The study also searched three major governmental agencies—the Korean Presidential Office Broadcast (KTV), Korea Disease Control and Prevention Agency (KDCA), and KMHW—following both website and non-website communications regarding COVID-19 vaccination recommendations. Any communication material used to inform people—official documents, daily briefings, news reports, and website information and announcements—was collected as primary source data for the study. Additionally, a review of local media reports on COVID-19 vaccination was conducted using the online database Bigkind through keyword search.

## Results

The present study found that the social marketing public health campaign targeting COVID-19 vaccination behavior effectively increased voluntary uptake of the COVID-19 vaccine in Korea. The intended behavioral outcomes were observed when social marketing techniques were adopted in the immunization campaign. In August 2022, Korea achieved high COVID-19 immunization coverage. About 94.8% of Korean citizens over the age of 12 years had completed their second dose; 71.3% had received the third (booster) dose; and 16.2% (over the age of 50) had completed the fourth dose (KMHW, 2022a). The pre-campaign survey revealed that 67.7% of Koreans had concerns about vaccine safety and side effects and preferred to wait and see how others responded to it (Dailymedi News, 2021). However, a high prevalence of vaccine hesitancy, delay, or refusal was significantly decreased and behavior adaptation rates increased when people were exposed to social marketing immunization campaign messages. This change indicates that social marketing communication techniques effectively responded to concerns in the public sector, successfully engaged with the target audience’s perceptions, and changed their attitude, leading to action.

## Discussion

### Korea’s COVID-19 vaccine communication strategy

Korea was among the first countries to be severely affected by COVID-19 due to its proximity to China. The first confirmed case was reported on January 20, 2020, followed by three waves of infection. The first peak was reached between February and March 2020, the second between August and September 2020, and the third between November 2020 and February 2021 (KMHW, 2020a). In February 2021, Korea announced a COVID-19 vaccination program, aiming to immunize 70% of the adult population by September 2021 (KDCA, 2021a, 2021b). However, most Koreans were reluctant to take the vaccine because of uncertainties about adverse long- or short-term side effects, and lack of awareness. Understanding the public’s concern and general attitude toward COVID-19 vaccines, the Korean public health authority started a social marketing campaign to educate the public regarding vaccine safety and efficacy. The program gradually increased vaccine confidence among the general public and engagement behaviors were manifested. The country’s slow initial vaccination uptake progressively improved as the promotion campaign continued, and a high uptake rate was eventually achieved. By August 2022, 94.8% of Korean citizens over the age of 12 had completed the second dose, 71.3% had received the third (booster) dose, and 16.2% (those aged over 50) had received the fourth dose (KMHW, 2020a).

#### Product communication

Whenever faced with hesitancy or resistance, the Korean public health authority provided detailed information to build public trust in vaccines (products) (KDCA, n.d.-a). Specifically, they explicitly stated the need for vaccination uptake (behavior change), stressing its worth and the multifold benefits of COVID-19 immunization. Great values of COVID-19 vaccines include high efficacy; protection from developing severe symptoms, hospitalization, and death; prosocial behavior for the community; and reconnecting with social networks without concern of infection (KDCA, n.d.-a; KMHW, n.d.). Such a goal is attainable if each individual adopts protective behaviors and takes the COVID-19 vaccine (Statistic Korea, n.d.-b).

The KDCA presented scientific evidence-based communication about the benefits of vaccination during its daily public briefings, informing the public about clinical trial results that suggest high vaccine efficacy rates (Polack et al., 2020; Voysey et al., 2021). The KDCA addressed the benefits of booster shots to stay protected against subvariants by showing how people who had taken the third booster had reduced the risk of infection and hospitalizations compared with those who received only two doses (KMHW, 2022b). Communicating proven scientific data to the public helped individuals better understand the vaccine’s benefits and perceive immunization as a self-protective behavior.

Extending the values of immunization beyond individual health benefits, the Korean government has publicized the significant social benefits of vaccination to appeal to the public; as herd immunity is achieved, population immunity against pathogens can be attained. In this way, countries can protect the lives of their citizens and the most vulnerable groups and escape the tremendous social and economic burden of COVID-19. Thus, failing to curtail virus transmission greatly risks national security. Owing to the collectivistic nature of Korean society, where people tend to value the community and their common interests over individual needs, this was a convincing strategy (KDCA, 2021c; Hong, 2022). Consequently, the public made informed decisions. In October 2021, the nation’s herd immunity threshold was surpassed; 41.31 million Korean adults (79.7% of the total population) had completed their COVID-19 immunization (Our World in Data, 2022). A post-vaccination survey revealed that the public’s primary reasons for COVID-19 immunization were to protect their family members (76.4%) and to help the nation achieve herd immunity (63.9%) (Yonhap News, 2021).

During the rollout, the Korean government introduced incentives to support the behavior change (*Hug*). Benefits such as exemptions from quarantine rules, paid-vaccine holidays, free access to parks, free lodging in private resort facilities, and free meals in certain restaurants across the country were provided to vaccinated individuals (Korean Ministry of Culture, Sports, and Tourism [KMCST], 2021).

#### Price communication

Barriers can be linked to various factors, such as values, knowledge, abilities, and economic standing. However, the most common and significant obstacle to vaccination is associated with risk perception, such as side effects. Although the COVID-19 vaccine was acknowledged as the most effective public health intervention for virus control, its adverse effects have been regarded as a major barrier to vaccine intention (Nguyen et al., 2021). As stated previously, safety and potential adverse events were the primary barriers among the Korean public regarding the COVID-19 vaccination. To reduce the psychological price/cost of vaccination, the KDCA provided advocative proof of the safety and adequacy of COVID-19 immunization. In their briefings, the KDCA shared their recommendation of the importance of undertaking the COVID-19 vaccine and offered evidence from trial data, suggesting vaccine-attributable severe side effects were extremely rare, mostly short-term injection site pain, tiredness, and mild headaches (KDCA, 2021c).

The KDCA’s public message also focused on fostering vaccine literacy (e.g., how mRNA/protein subunit vaccines work to produce viral proteins and develop immunity in the body, active ingredients, effectiveness against variant strains of COVID-19, up-to-date clinical trial outcomes, and side effects of vaccines (KDCA, n.d.-a). To tackle misinformation that creates negative public opinion, the Korean government widely employed fact-checking and debunking strategies against anti-vaccination propaganda on various social media. If invalidated misinformation is circulated and receives public attention, the public health authority promptly refutes the rumors via fact-checking and debunking.

The government aimed to reduce the target behavior’s costs/prices/barriers; it also increased the costs of the competing behavior by employing disincentive measures. They announced compulsory quarantine regulation for the contact between confirmed COVID-19 cases and inbound travelers, and a 14-day mandatory quarantine was applied for unvaccinated individuals (The Korea Herald, 2021). People had to prove their negative status with a negative PCR test before being released from isolation (*Shove*). On December 13, 2021, the Korean government introduced the “vaccine passport” to further increase barriers against non-compliant behavior (KDCA, n.d.-a). During this period, adults had to present a vaccine certificate or a negative PCR test to access public venues such as gyms, concert halls, cinemas, and hospitals (*Smack*). This regulation was abolished on March 1, 2022, because of strong public opposition. Nevertheless, the COVID-19 certificates helped the country control the COVID-19 pandemic and maintain behavior adoption after reaching herd immunity.

#### Place communication

Throughout the immunization process, the Korean government provided open and honest communication to the public and widely disseminated information regarding COVID-19 vaccination via diverse media channels. Such information included how to make reservations, where to get vaccinations, and access to separate queues for older adults during vaccination registration (KDCA, n.d.-b, n.d.-d). Regarding the booking process, the KDCA made multiple formats available to ensure quick and easy public access. The KDCA’s website was the primary booking method; it provided a clear, easy-to-understand format for people to follow.

In addition, telephone options and in-person reservations were made available for older adults who were not familiar with the internet. The campaign message also informed the public about the accessibility of the vaccine and vaccination locations in real-time. In May 2021, the KDCA announced the availability of mobile app services on Naver and KaKao Talk messenger. These services enabled the public to search for nearby vaccination centers and provided up-to-date information on vaccine data. The government carefully selected the physical sites to ensure easy and convenient venues. Large sports arenas, cinemas, conference halls, hospitals, and clinics were available, along with free vehicle pick-up and drop-off services for individuals in remote areas (KMHW, 2020b). The government used clear, multiple, easy-to-read signs to direct people to vaccination centers (especially older adults). In the centers, trained staff guided them through the vaccination services and addressed their concerns. They also provided special care and friendly guidance for older adults.

#### Promotion communication

There were three key messages in the Korean government’s COVID-19 vaccination promotion campaign: vaccine services were free, vaccines were safe and effective, and vaccines provided the best protection against the virus for individuals, their families, and the community (Statistic Korea, n.d.-a). The goal of the promotional message was to persuade the public that they would benefit more if vaccinated. In January, a month before the rollout, the KDCA stated that vaccines would be free for all Korean citizens and foreign residents. This promotion strategy removed financial barriers and facilitated uptake behavior.

For successful immunization against COVID-19, the Korean public health authority conducted an extensive public education campaign concerning the safety and efficacy of vaccines with rational arguments. In this process, they recruited credible healthcare professionals, religious and community leaders, and local celebrities who had established trusting relationships with the community. Their advocacy message about vaccine safety and efficacy could support the message of public health communication (Catholic Bishop’s Conference of Korea, 2021).

Finally, the government’s promotional message advertised the benefits of behavior change (vaccination) as the best protection for oneself, others, and the whole nation (Statistic Korea, n.d.-b). In collectivistic cultures such as Korea, prosocial messages appeal to the sense of social responsibility and are more effective in engaging the public emotionally to work toward a common goal.

To reach the entire nation, the government communicated through the media. They used numerous conventional and emerging social media outlets, including official media and YouTube, Facebook, Twitter, Naver, and Kakao. The following information channels play an important role in increasing public trust in the safety and effectiveness of vaccines by disseminating current vaccine research development throughout immunization campaigns: mass media (TV, radio, Internet, metro/bus posters, street banners and signs, electronic documents, etc.), public videos (hospital waiting areas, public facilities, local city offices, community service centers, etc.), service-based communication (doctors, nurses, and healthcare workers), special events, and advocacy via influencers (political leaders, public health officials, local community and religious leaders, local celebrities, etc.) (KDCA, 2018). To further increase the urgency of vaccine uptake, the Korean government sent reminders via text messages, pamphlets, and other informational materials to capture public attention (*nudge*). Sending repeated messages, notifying people of the vaccination schedule, and prompting implementation reminded the public to get vaccinated.

### Campaign effect: increasing the COVID-19 vaccine acceptance

Social marketing is a proven behavioral change technique, widely adopted in public health campaigns for promoting changes in knowledge, norms, belief, attitude, and behavior of the general population (Lee et al., 2022; Melovic et al., 2020; Coffie et al., 2022; Evans and French, 2021; Osborne et al., 2021). In the context of COVID-19 vaccination, the current study found that the social marketing vaccination campaign was effective in increasing individuals’ intention to adopt immunization behaviors as it could significantly improve the targets’ knowledge, belief, attitude, and barriers to COVID-19 vaccination (Adane et al., 2022; Salali et al., 2022; Evans and French, 2021).

#### Knowledge, belief, and attitude toward COVID-19 vaccines

The level of vaccine hesitancy significantly decreased in Korea due to the ongoing efforts of Korean public health officials to convince and educate the public about the safety and efficacy of COVID-19 vaccines. In the pre-vaccination period, a significant portion of the Korean population was hesitant to vaccinate (Dailymedi News, 2021; Views and News, 2021). Only a small portion of people indicated a positive intention to vaccinate (Heo, 2021; You, 2021; Lee and Yang, 2021). Importantly, these studies reveal that high rates of vaccine hesitancy and skepticism are associated with safety concerns, insufficient vaccine literacy (e.g., poor perception or low belief in the efficacy of vaccines), and lack of awareness about the threat of COVID-19.

To our knowledge, the Korean population’s overall perception, knowledge, and attitude toward COVID-19 vaccination have become significantly favorable as the vaccination campaign progressed. A previous study also suggested that there were substantial improvements in overall knowledge, perceptions, and attitudes toward COVID-19 vaccination among the Korean public during the vaccine rollout, indicated by a steady increase in vaccine acceptance (Choi et al., 2022). A survey by Choi et al. (2022) conducted during the first rollout in March 2022 indicates an improved perception toward vaccines. After months of public campaigns on COVID-19 vaccination, the majority of the Korean public (75.5%) felt that the decision to vaccinate was important and worth it. Moreover, 74.3% of people said they believed in the vaccine’s efficacy, indicating high vaccine intention (You, 2021). This increased vaccine confidence and their positive attitudes could lead them to have voluntary behavioral engagement toward vaccination.

#### Perceived barriers toward COVID-19 vaccines

The study observed that the social marketing immunization campaign effectively reduced perceived barriers to vaccination. As noted, the Korean public was skeptical regarding vaccine safety and its side effects (You, 2021; Lee and Yang, 2021). However, the public’s perception of barriers was significantly reduced, and their risk perception toward vaccination diminished during the COVID-19 immunization campaign. On social media platforms, keywords such as “side effects” and “safety” (the most frequent terms on the top four platforms, Naver, Daum, Google, and Twitter, during pre-vaccination in Korea) received less attention after the public immunization campaign began. Negative keywords related to the side effects associated with COVID-19 vaccines (e.g., blood clots, severe allergic reactions, or death) did not appear either (Choi et al., 2022). This observation was supported by a national survey conducted in April 2021, suggesting that the Korean public’s perceived benefit of vaccines surpassed the perceived barriers in all age groups. This tendency was reported to be even greater among older adults (60+ years) (You, 2021).

### Five effective public health messages to boost vaccine intention

Overcoming the COVID-19 pandemic necessitates adequate behavioral changes. Communicating effectively and persuasively with the public is critical to elicit a behavioral response (Betsch, 2020). This section discusses how the Korean government framed campaign messages to maximize their message effectiveness, persuading the general population to adopt vaccination behavior and identifying the main characteristics of the campaign, such as high proactiveness, credibility, fighting misinformation, emphasizing social norms, and coherence of official communication.

(i) *Proactiveness*: Delivering critical vaccine information to the public during a pandemic could create confusion and concern (Lyu et al., 2022). Hence, public health communication must anticipate problems and share necessary information. If the target audience’s attention is not captured from inception, misinformation and fake news by anti-vaccine propagandists will impact the process (Loomba et al., 2021; Roetzel, 2019; Zheng, 2022). Given the enormous amount of negative information about vaccines on social media, a negative attitude toward vaccination is likely to develop, resulting in low immunization uptake rates (Olson, 2020; Kim et al., 2021). Thus, proactive communication interventions have been emphasized and implemented as effective communication strategies throughout Europe (Butler et al., 2015). Korea also adopted the “Be First” messaging principle, emphasizing proactive communication in responding to public health emergencies (KDCA, 2018). Striving to be the louder voice in the country as the official information channel regarding COVID-19 vaccines, the country could successfully overcome vaccine hesitancy. Consequently, by proactively hushing the misinformation and anti-scientific attitudes, the Korean public health authority decreased anxieties and skepticism about vaccine safety and efficacy (Choi et al., 2022), and effectively increased vaccination uptake (KDCA, n.d.-c).

(ii) *Credibility*: Research has shown that higher credibility will likely generate greater message compliance (De Meulenaer et al., 2018). Credibility is a central feature of effective and persuasive health communication. Perceiving a source as trustworthy and valid can significantly affect the beliefs and intentions of the target population to adopt the suggested behaviors. Vaccine hesitancy can considerably decrease when the public perceives the public health authority as trustworthy (Trent et al., 2022). Korea’s public healthcare system is well equipped to provide quality healthcare services; therefore, public trust in the system is already high. During the COVID-19 pandemic, the Korean public showed a significantly high level of trust in the KDCA, which led to the pandemic mitigation plans and national inoculation policies (78% in the first week of June 2021) (Korea Research, 2021). Vaccine hesitancy or refusal can increase when the public loses faith in the governmental authority. A higher degree of confidence in public health authorities rendered the Korean public less prone to fake news or conspiracy theories. People were more willing to listen to the immunization messages delivered by Chong En-Kyong, the KDCA’s key commissioner, and other well-known public health scholars and expert groups (Hong, 2022; Abu-Akel et al., 2021). We may infer that a high level of public confidence in government organizations increases the likelihood of message acceptance toward COVID-19 vaccines and decreases the perceived validity of fake news. Therefore, conspiracy theories may not significantly affect Koreans’ views and attitudes toward vaccination.

(iii) *Fighting misinformation*: Misinformation about COVID-19 vaccines can cause public vaccine hesitancy or refusal by arousing doubt, anxiety, and concerns about vaccines (Nguyen et al., 2021; Loomba et al., 2021; Springer and Özdemir, 2022). The Korean government used fact-checking and debunking strategies to tackle mis/disinformation and vaccine hesitancy, and discredit commonly held myths; they exposed logical flaws in the misinformation to correct any misconceptions (e.g., COVID-19 vaccines cause infertility and other diseases, they have a microchip, they cause death, they alter one’s DNA, etc.) (KDCA, 2021d). Concurrently, the KDCA and Korea Communication Commission (KCC) introduced a reporting system on the website www.KCC.go.kr/vaccinejebo to prevent the spread of misinformation on vaccine efficacy and safety. All anti-vaccine messages were monitored using artificial intelligence (AI), and messages spreading misinformation, fear, and negative opinions regarding government intervention were reported on the website and deleted from the media platform (KCC, n.d.). To further reduce the effect of fake news on immunization, Twitter, Korea, and YouTube removed over 43,000 tweets and 1,000,000 erroneous COVID-19-related messages and videos that disseminate false claims about vaccines (WHO, 2022b). This strategy significantly reduced public exposure to misinformation, which could impact vaccine intention.

(iv) *Emphasizing social norms and prosocial behavior*: Perceived social norms and beliefs about how others conduct themselves impact a person’s behavior and intent (Schultz et al., 2007). In the context of COVID-19 vaccination, individuals are more inclined to follow the COVID-19 protocol and get vaccinated if they believe that more people are participating in these preventive actions (Rabb et al., 2022). Notably, a study on the Korean public’s awareness and attitude toward COVID-19 immunization corroborates this result, showing a shift in perception from negative to positive as more people get the inoculation (Choi et al., 2022). We can infer that people’s beliefs and intentions toward COVID-19 vaccines are affected by the higher vaccination intentions and behaviors of others.

In particular, people belonging to collectivistic cultures prioritize the interests of society over the individuals’ interests. Preferences are more likely to be affected by beliefs about other people’s intentions—social norms—within the community and their close social networks, such as family, friends, and neighbors (Salali et al., 2022; Cammett and Lieberman, 2020). During the vaccine rollout, the Korean public health authority effectively influenced people’s vaccination behavior and intention. They used messages such as “everyone gets vaccinated,” “taking the vaccine is the right thing to do,” “get vaccinated to return to normal life,” “achieve herd immunity with the vaccination,” and “protect your family elders with the COVID-19 vaccine,” to assert social norms and prosocial behavior (KDCA, 2021c; KDCA, n.d.-c). The Korean government appealed to its people’s collective responsibility and intrinsic motivation to avoid harm to their social ties, thereby aligning its population with the public health goal of achieving herd immunity within a year. These messages from public health authorities about the country’s new immunization goal gave the public the collective task of vaccination against COVID-19 as early as possible. This message significantly affected vaccine intention and uptake behavior among the Korean public (Hong, 2022).

(v) *Coherence*: Previous studies suggest that clear, coherent, and consistent public health messages about the safety and efficacy of vaccines can effectively decrease public confusion and anxiety about vaccination, and increase vaccination intention (Jin et al., 2021; Murewanhema et al., 2022; Hyland-Wood et al., 2021). In the context of COVID-19 immunization communication, Korean public authorities sought to increase message receptivity and public support for immunization, reducing belief in the infodemic about COVID-19 vaccines (KDCA, 2018).

Since the rollout began, the KDCA provided clear and specific instructions to the public, calling for action (e.g., “To register for the vaccine, visit the KDCA website”; or “call the 1339 help desk directly”) and particular events (e.g., “From February 6 to April 2021, healthcare professionals, older adults over 75 years of age, and people with chronic conditions have to be vaccinated”) (KDCA, 2021a). Communicating coherent and consistent pro-vaccine messages was used to prompt people to adopt healthy attitudes and vaccination behaviors (e.g., “COVID-19 vaccines provide the best protection against novel coronavirus,” “The benefits of getting a COVID-19 vaccine outweigh the risks”) (KDCA, 2021c). All public health figures and organizations in Korea (e.g., the Central Disaster and Safety Countermeasure Headquarters, KMHW, KDCA, and local governments) applied this coherent and consistent message strategy to their vaccination messages (KMHW, 2019). Korea was able to communicate more effectively with the public and boost their vaccination intentions by presenting a uniform and united front among all government agencies throughout the vaccination campaign.

## Conclusions and outlook

Government communication could play a substantial role in influencing public attitudes toward immunization. The public’s understanding of the pandemic, immunization, and vaccine intentions can be shaped by public health messages. This study analyzed the Korean government’s COVID-19 mass vaccination campaign strategies and interventions and offered insights contributing to global discussions on health communication strategies and approaches. The results indicate that a robust social marketing campaign can effectively customize messages according to the target population’s interests and values, persuade the public about the product (the need for vaccination), and overcome barriers to immunization acceptance. It enhances general confidence in the COVID-19 vaccine and, at least partly, overcomes hesitancy by increasing and reinforcing vaccine literacy, providing balanced information about the benefits and risks, and dispelling rumors and misconceptions. Furthermore, the study identified five key communication attributes—proactiveness, credibility, fighting misinformation, emphasizing social norms and prosocial behavior, and coherence—for official communication to improve communication interventions for maximum effect. These attributes can be applied in other countries’ vaccine messaging campaigns and national immunization programs. Although the study has explored the link between government communication and individuals’ intention to vaccinate through a social marketing perspective, it has limitations. This study relied on a document analysis design. Future studies must employ a quantitative design, such as a cross-sectional survey or regression analysis, to further examine the causal effects between variables. In addition, this study was conducted in Korea, and the results should not be generalized to other cultural settings. Consequently, future research must examine communication and messaging strategies from other geographic locations with more diverse population groups and sociocultural contexts. Repeated local studies using other approaches that facilitate vaccination will be essential for vaccine promotion. This is essential for public health policymakers to devise suitable communication intervention strategies to improve vaccination, booster uptake, and future immunization.
